# Acute cardiovascular effects of controlled exposure to dilute Petrodiesel and biodiesel exhaust in healthy volunteers: a crossover study

**DOI:** 10.1186/s12989-021-00412-3

**Published:** 2021-06-14

**Authors:** Jon Unosson, Mikael Kabéle, Christoffer Boman, Robin Nyström, Ioannis Sadiktsis, Roger Westerholm, Ian S. Mudway, Esme Purdie, Jennifer Raftis, Mark R. Miller, Nicholas L. Mills, David E. Newby, Anders Blomberg, Thomas Sandström, Jenny A. Bosson

**Affiliations:** 1 Department of Public Health and Clinical Medicine, Section of Medicine, Umeå University, Umeå, Sweden; 2 Department of Surgical Sciences, Uppsala University, Uppsala, Sweden; 3 Thermochemical Energy Conversion Laboratory, Umeå University, Umeå, Sweden; 4 Department of Materials and Environmental Chemistry, Stockholm University, Stockholm, Sweden; 5 MRC-PHE Centre for Environment and Health, NIHR Health Protection Research Unit in Environmental Exposures and Health, Imperial College London, London, UK; 6 Centre for Inflammation Research, University of Edinburgh, Edinburgh, UK; 7 University/BHF Centre for Cardiovascular Science, University of Edinburgh, Edinburgh, UK; 8 Usher Institute of Population Health Sciences and Informatics, University of Edinburgh, Edinburgh, UK; 9 Dept. of Medicine, Division of Respiratory Med, University Hospital, 90185 Umeå, Sweden

**Keywords:** Air pollution, Particulate matter, Diesel, Biodiesel, Cardiovascular system, Vascular function, Vasomotor dysfunction, Thrombosis, Endothelial function

## Abstract

**Background:**

Air pollution derived from combustion is associated with considerable cardiorespiratory morbidity and mortality in addition to environmental effects. Replacing petrodiesel with biodiesel may have ecological benefits, but impacts on human health remain unquantified.

The objective was to compare acute cardiovascular effects of blended and pure biodiesel exhaust exposure against known adverse effects of petrodiesel exhaust (PDE) exposure in human subjects.

In two randomized controlled double-blind crossover studies, healthy volunteers were exposed to PDE or biodiesel exhaust for one hour. In study one, 16 subjects were exposed, on separate occasions, to PDE and 30% rapeseed methyl ester biodiesel blend (RME30) exhaust, aiming at PM_10_ 300 μg/m^3^. In study two, 19 male subjects were separately exposed to PDE and exhaust from a 100% RME fuel (RME100) using similar engine load and exhaust dilution. Generated exhaust was analyzed for physicochemical composition and oxidative potential. Following exposure, vascular endothelial function was assessed using forearm venous occlusion plethysmography and ex vivo thrombus formation was assessed using a Badimon chamber model of acute arterial injury. Biomarkers of inflammation, platelet activation and fibrinolysis were measured in the blood.

**Results:**

In study 1, PDE and RME30 exposures were at comparable PM levels (314 ± 27 μg/m^3^; (PM_10_ ± SD) and 309 ± 30 μg/m^3^ respectively), whereas in study 2, the PDE exposure concentrations remained similar (310 ± 34 μg/m^3^), but RME100 levels were lower in PM (165 ± 16 μg/m^3^) and PAHs, but higher in particle number concentration. Compared to PDE, PM from RME had less oxidative potential. Forearm infusion of the vasodilators acetylcholine, bradykinin, sodium nitroprusside and verapamil resulted in dose-dependent increases in blood flow after all exposures. Vasodilatation and ex vivo thrombus formation were similar following exposure to exhaust from petrodiesel and the two biodiesel formulations (RME30 and RME100). There were no significant differences in blood biomarkers or exhaled nitric oxide levels between exposures.

**Conclusions:**

Despite differences in PM composition and particle reactivity, controlled exposure to biodiesel exhaust was associated with similar cardiovascular effects to PDE. We suggest that the potential adverse health effects of biodiesel fuel emissions should be taken into account when evaluating future fuel policies.

**Trial registration:**

ClinicalTrials.gov, NCT01337882/NCT01883466. Date of first enrollment March 11, 2011, registered April 19, 2011, i.e. retrospectively registered.

**Supplementary Information:**

The online version contains supplementary material available at 10.1186/s12989-021-00412-3.

## Background

Particulate matter air pollution (PM) has a well-established and consistent association with cardiovascular and respiratory morbidity and mortality [1–3]. This link has proven strongest for fine combustion-derived PM (particle diameter < 2.5 μm; PM_2.5_) [4]. Exhaust from diesel engines is an important contributor to PM_2.5_ in urban environments, as well as being a major source of smaller combustion-derived nanoparticles [5]. Time in traffic has been associated with triggering of acute myocardial infarction [6] and there is a wealth of data from controlled human experimental exposure studies showing that petrodiesel exhaust (PDE) has multiple adverse effects throughout the cardiovascular system [7–10].

Due to adverse environmental effects of fossil fuels, considerable efforts have been made to find alternative renewable energy sources. The European Union has a current target for 10% use of biofuels within transportation (EU Directive 2009/28/EC), Finland targets 30% biofuel by 2030 [11] and similar efforts are planned in the US, China, India and elsewhere [12].

The ideal biofuel would be cheap to produce, compatible with existing infrastructure and engine technologies, but, importantly, would not cause harm to either human health or the environment [13]. Biodiesel (diesel fuel made from biological substances rather than petroleum sources) meets several of these criteria and, as a result, it is increasingly added to petrodiesel used in commercial and industrial settings [14, 15].

Untreated oils from vegetables and plants are not suitable to run in modern diesel engines without a trans-esterification process. When rapeseed oil is used, the end product is rapeseed methyl ester (RME), which has good ignition and lubrication properties. RME is popular in Europe as it can be produced locally, blended into petrodiesel and used in existing diesel engines. Novel production methods of biodiesel are being developed to decrease land usage and increase yield, however, currently rapeseed and soybean are the major crops for biodiesel production in Europe and the US, respectively.

While the efficiency of biofuel combustion has been well-studied, relatively little focus has been directed towards the health effects of emissions generated from biofuels, despite the rapid introduction of these fuels in societies and calls for such research [14, 16]. Several in vitro studies and animal studies have addressed the potential health effects of biodiesel exhaust exposure [17–19], but, to our knowledge, there is only one human exposure study in a mining environment reporting on respiratory and peripheral blood inflammatory markers [20].

In the present study, we assessed the potential acute vascular and thrombotic effects of replacing petrodiesel with a 30% or 100% rapeseed biodiesel formulation, following controlled exposure in young healthy volunteers. It was hypothesized that inhalation of exhaust from biodiesel formulations would induce lesser cardiovascular effects compared to standard petrodiesel fuel.

## Results

Sixteen subjects completed study one (RME30 vs. PDE exposure and 19 subjects completed study two (RME100 vs. PDE exposure) (Table 1). The studies were well tolerated by all subjects with no adverse events. There were no significant differences in lung function measured as VC and FEV_1_ pre vs. post exposure (data not shown).

**Table 1:** Subject characteristics

Parameter	Study one, ***n*** = 16, 2 females.		Study two, ***n*** = 19, all males	
**Age, years**	25 (21–29)		28 (20–38)	
**Height, cm**	Males: 181 (173–195) Females: 171 (166–176.5)		181 (168–190)	
**Weight, kg**	Males: 75 (67–88) Females: 69 (60–79)		79 (55–97)	
**BMI, kg/m**^**2**^	Males: 23 (20–25) Females: 23 (21–25)		24 (19–29)	
**Pre exposure measures**	**Petrodiesel**	**RME30**	**Petrodiesel**	**RME100**
**Vital capacity, L**	5.8 ± 0.3	5.9 ± 0.3	6.3 ± 0.3	6.3 ± 0.3
**FEV**_**1**_**, L**	4.6 ± 0.2	4.6 ± 0.2	4.8 ± 0.2	4.8 ± 0.2
**FE**_**NO**_ **50, ppb**	12 ± 1	11 ± 1	13 ± 1	15 ± 2
**Systolic blood pressure, mm hg**	121 ± 3	123 ± 2	136 ± 3	135 ± 3
**Diastolic blood pressure, mm hg**	69 ± 1	71 ± 1	79 ± 2	78 ± 2
**Heart rate, bpm**	65 ± 2	64 ± 2	68 ± 2	67 ± 2
**Hemoglobin, g/L**	148 ± 3	147 ± 3	151 ± 2	151 ± 2
**Leukocyte particle concentration × 10**^**9**^**/L**	5.7 ± 0.3	5.7 ± 0.4	5.4 ± 0.2	5.5 ± 0.3
**Platelet concentration, ×10**^**9**^**/L**	231 ± 11	236 ± 11	232 ± 11	230 ± 10
**Lymphocytes, ×10**^**9**^**/L**	2.1 ± 0.2	2.1 ± 0.2	2.2 ± 0.2	2.2 ± 0.2
**Monocytes, ×10**^**9**^**/L**	0.5 ± 0.0	0.5 ± 0.0	0.5 ± 0.0	0.5 ± 0.0
**Neutrophils, ×10**^**9**^**/L**	2.9 ± 0.2	3.0 ± 0.2	2.6 ± 0.1	2.6 ± 0.2

Median and range for anthropomorphic measures. Mean ± standard error of the mean (SEM) for pre-exposure baseline cardiorespiratory and hematological measures

### Exposures

In study one (RME30 vs. PDE), the comparison aimed at a target concentration in the exposure chamber of PM_2.5_ 300 μg/m^3^ for both fuel types. To account for the commonly observed lower PM mass emissions of biodiesel, study 2 (RME100 vs. PDE) was not designed for equal PM mass concentrations in the exposure chamber. Rather, the RME100 exposure was using similar engine and exhaust dilution settings as the PDE exposure (aimed at PM_10_ 300 μg/m^3^), which resulted in lower mass emissions of RME100 PM.

**Fig. 1:**
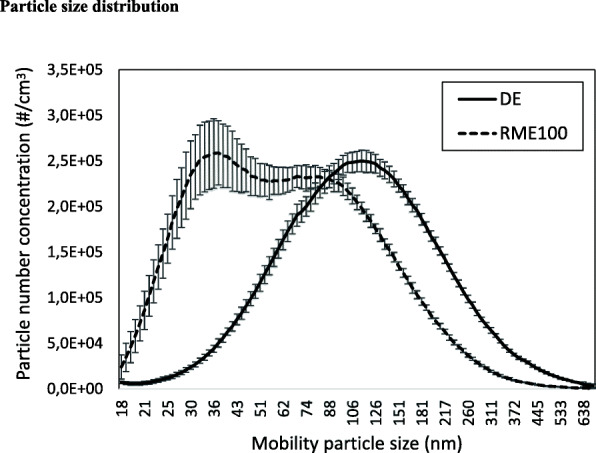
Particle size distribution during PDE and RME100 exhaust exposure. Mean with standard deviation. Exposures were kept constant and measurements (*n* = 3) were spread out over the exposure series

Basic characterization of RME30 exhaust was relatively similar to that of PDE, as regards PM_10_, total hydrocarbons and elemental carbon/organic carbon ratio (EC/OC ratio) (Table 2). Compared to PDE, RME100 exhaust contained more and smaller particles. (Table 2, Fig. 1). RME100 exhaust particles had a lower EC/OC ratio and less particle-bound polycyclic aromatic hydrocarbons (PAHs), as compared with PDE. The gas phase of RME30 and RME100 exhaust contained higher concentrations of NOx than PDE. The fact that NOx differed slightly between RME exposures, was due to minor tuning issues required by the engineers managing the exposure set up.

**Table 2:** Exposure characteristics

	Study one		Study two	
	Petrodiesel	RME30	Petrodiesel	RME100
**PM**_**10**_**, μg/m**^**3**^	314 ± 27	309 ± 30	310 ± 34	165 ± 16
**Total Hydrocarbons, ppm**	1.0 ± 0.1	0.9 ± 0.1	0.9 ± 0.2	0.9 ± 0.2
**NO**_**x**_**, ppm**	3.9 ± 1.0	4.6 ± 1.0	6.1 ± 0.4	7.3 ± 0.5
**NO**_**2**_**, ppm**	0.7 ± 0.1	0.3 ± 0.1	0.4 ± 0.1	0.5 ± 0.1
**Temp, C**	22 ± 1	22 ± 1	22 ± 2	22 ± 1
**Relative humidity, %**	36 ± 13	33 ± 11	28 ± 11	30 ± 13
**Particle number concentration ×10**^**5**^**/cm**^**3**^	n/a	n/a	1.7 ± 3.2	2.2 ± 1.4
EC/OC, ratio*	0.66 ± 0.01	0.64 ± 0.02	n/d	0.43 ± 0.03
**Particle bound PAHs**, **ng/m**^**3**^	156 ± 16	127 ± 15	n/d	76 ± 24
**Semi volatile PAHs, ng/m**^**3**^	244 ± 81	158 ± 37	n/d	225 ± 167

Exposure characteristics, mean and standard deviation. *Elemental (EC) and organic (OC) carbon. Semi-volatile PAHs are the sum of; phenanthrene, anthracene, 3-methylphenanthrene, 2-methylphenanthrene, 2-methylanthracene, 9-methylphenanthrene, 1-methylphenanthrene, 4*H*-cyclopenta [*def*] phenanthrene, 2-phenylnaphthalene, 3,6-dimethylphenanthrene, 3,9-dimethylphenanthrene, fluoranthene, pyrene, 2-methylpyrene, 4-methylpyrene, 1-methylpyrene, benz [*a*] anthracene, and chrysene. For Study one *n* = 16 and for study two *n* = 19, except EC/OC and PAHs *n* = 3 for studies one and two

**Fig. 2:**
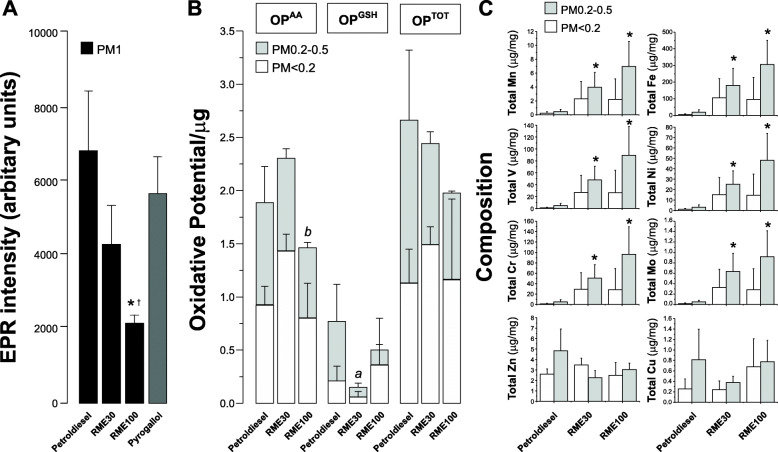
Oxidative potential and metal content of exhaust PM derived from petrol diesel and RME blended fuel combustion. Panel **a** shows superoxide free radical generation using electron paramagnetic resonance (EPR) with the spin-trap Tempone-H (1 mM). All particulates were suspended at an equivalent concentration of 0.1 mg/mL in physiological saline. Pyrogallol (0.1 mM) is used as a positive control to spontaneously generate superoxide. RME100 generated significantly less superoxide than petrodiesel (*p < 0.05) or RME30 (†*p* < 0.05) (unpaired t-tests, *n* = 6–10). Panel **b** represents ascorbate- and glutathione-dependent oxidative potentials (OPAA and OPGSH, respectively) for the PM < 0.2 μm and PM0.2–0.5 μm fractions are illustrated, with the data expressed per μg of extracted PM (*n* = 3, separate filters, per fraction and fuel type). A total aggregated OP (OP^TOT^) is also illustrated reflecting the sum of the OP^AA^ and OP^GSH^ measures. Data are illustrated as means with standard deviation, with comparison between groups performed on the sum of the OP for the two fractions combined using the students t-test (*P* < 0.05): ‘a’ petrol diesel vs, RME30; ‘b’ RME30 vs RME100; no significant differences were observed between petrol diesel and RME100. Panel **c** represents the concentration of a selection of the measured metals in both PM fractions derived from each fuel type (*n* = 3). Zn = zinc, Cr = Chromium, V = Vanadium, Mn = Manganese, Cu = Copper, Mo = Molybdenum, Ni = Nickel, Fe = Iron. Asterisks represent significant differences (*p* < 0.05) in concentration relative to petrodiesel. No significant differences were noted between the fuel types in the PM < 0.2 μm fraction and metal concentrations did not differ between the two fractions under each condition

The oxidative potential (OP) of PM measured with electron paramagnetic resonance (EPR) showed a lower oxidative potential with increasing RME content within the fuel. The RME100 fuel sample generated significantly less superoxide free radicals per mass of PM than the petrodiesel or RME30 (Fig. 2). Oxidative potential (OP) was also measured by the quantification of antioxidant losses from a synthetic respiratory tract lining fluid. This generated two metrics, based on ascorbate and glutathione oxidative consumption over a 4-h incubation period, OP^AA^ and OP^GSH^ respectively, expressed per unit mass (μg) of extracted PM. These data are illustrated in Fig. 2, panel a, for each fuel type and for the individual < 0.2 and 0.2–0.5 μm fractions. To allow comparison with the EPR data, the OP^AA^ and OP^GSH^ values have been aggregated to provide a total oxidative potential (OP^TOT^) across both fractions, which was equivalent to the sample employed for the EPR analysis. This composite measure of oxidative potential showed a similar overall pattern to that observed by EPR, with decreasing activity as the proportion of RME increased. The individual ascorbate and glutathione dependent OPs provided a less coherent pattern, with RME 100 having the lowest OP^AA^ and RME30 the lowest OP^GSH^ activities.

Of the metals considered, there were significantly higher levels of manganese (Mn), iron (Fe), vanadium (V), nickel (Ni), chromium (Cr) and molybdenum (Mo) in the RME30 blend and pure RME100, relative to the PDE in both the PM_< 0.2_ μm and PM_0.2–0.5_ μm size ranges (Fig. 2, panel b). The concentrations of the remaining metals/metalloids measured in each fuel fraction are summarised in Supplementary Table S1.

### Vascular and thrombosis studies

**Fig. 3:**
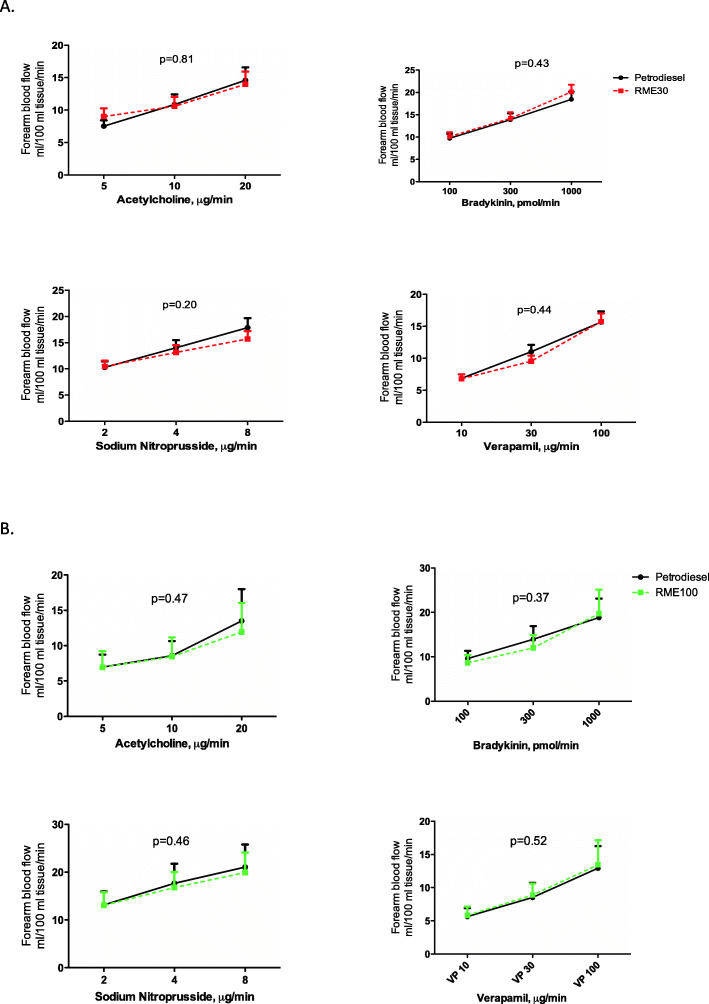
Forearm blood flow during intrabrachial infusion of vasoactive drugs 4–6 h post exposure, mL/100 mL tissue. Mean with 95% CI. The graph shows response to incremental doses of acetylcholine, bradykinin, sodium nitroprusside and verapamil following respective exposure. All vasodilators caused an increase in blood flow (*p* < 0.05 for all) that was similar between exhaust exposures (*p* > 0.05 for all). *P* values in the graph for respective vasodilator response in the infused arm following exposure to RME30 exhaust compared to PDE (*n* = 15) and RME100 exhaust compared to PDE (*n* = 18), 2-way ANOVA

Plethysmographic data and net t-PA release were determined as described previously [21, 22]. Infusion of all vasodilators generated a dose-dependent increase in forearm blood flow (2-way ANOVA for incremental doses of acetylcholine, bradykinin, sodium nitroprusside and verapamil, *p* < 0.05 for all drugs following all exposures). The vasodilatation was similar in both studies comparing responses for the respective agonists following PDE vs. RME30 (Study 1; *n* = 15) and PDE vs. RME100 (Study 2; *n* = 18) (2-way ANOVA comparing each drug in incremental doses following respective exposure; *p* > 0.05 for all, Fig. 3. A filtered air arm could not be included in the present study, due to the extensive logistical aspects of mounting three-arms studies with bilateral arm canulations and infusions. However, the consistent and very repeatable attenuation of vasodilatation by PDE vs. filtered air, based on pooled historical data from four studies [23–26] is given in Fig. S1 in the supplement.

**Fig. 4:**
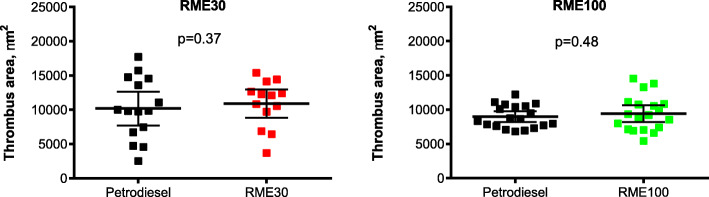
Ex vivo thrombosis formation in a model of acute arterial injury. Individual data points for mean thrombus area for each individual, line for group mean with 95% CI. There were no significant differences between RME30 and PDE (study one) or RME100 and PDE (study two) RME30 vs. PDE *p* = 0.37, *n* = 13 and RME100 vs. PDE *p* = 0.48, *n* = 19. P values from paired Student’s t-test

In both studies, ex vivo thrombus formation was similar following all exposures with mean total thrombus area in the perfusion chamber being 10,192 (95% CI [7718, 12,665]) μm^2^ following PDE and 10,888 (95% CI [8815, 12,922]) μm^2^ following RME30 exhaust in study one (*n* = 13), and 8973 (95% CI [8175, 9771]) μm^2^ following PDE and 9424 (95% CI [8194, 10,654]) μm^2^ following RME100 exhaust in study two (*n* = 19) (*p* = 0.37 and 0.48, respectively, Fig. 4).

**Fig. 5:**
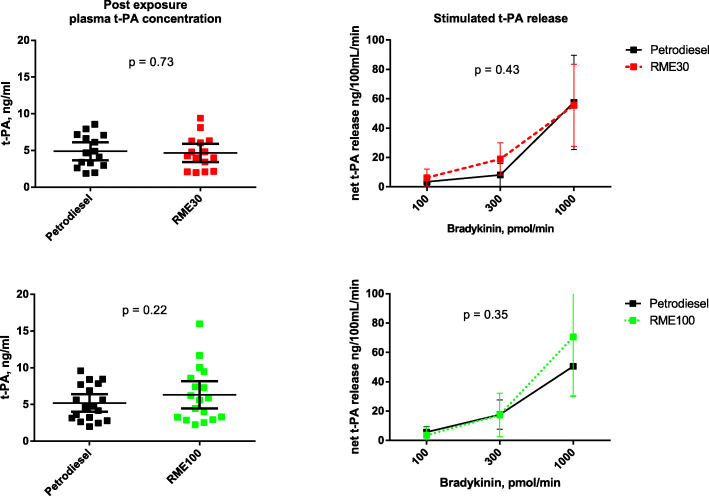
Mean plasma t-PA antigen concentrations after exposure to petrodiesel vs. RME 30 (upper left panel) and petrodiesel vs. RME100 (lower left panel) were not significantly different between exposures (*p* = 0.73 and *p* = 0.22, respectively). Stimulated release of t-PA plasma antigen concentrations following incremental doses of bradykinin infusions, expressed as nanogram per 100 mL tissue per minute, mean with 95% CI (upper and lower right panels). Bradykinin infusions caused dose dependent increases in t-PA antigen concentrations that were significant at *p* < 0.01 level for all exposures; petrodiesel and RME 30 (upper right panel) as well as petrodiesel and RME 100 (lower right panel) by 2-way ANOVA. The bradykinin stimulated increases in t-PA did not differ between petrodiesel or RME exposures (*p* = 0.43 and *p* = 0.35, respectively).

In both studies, mean plasma t-PA antigen concentrations were similar after all exposures, with 4.9 (95% CI [3.7, 6.1]) ng/mL following PDE and 4.7 (95% CI [3.4, 5.9]) ng/mL following RME30 exhaust in study one (*n* = 14), and 5.2 (95% CI [4.0, 6.4]) ng/mL following PDE and 6.3 (95% CI [4.5, 8.2]) ng/mL following RME100 exhaust in study two (*n* = 17) (Fig. 5, left panel). Bradykinin infusion caused a dose dependent release of t-PA that was similar following all exposures to PDE and RME, with no significant differences between the paired exposure scenarios (*p* = 0.43 for PDE vs. RME30 and *p* = 0.35 for PDE vs. RME100; 2-way ANOVA) (Fig. 5, right panel).

### Blood cell counts and platelet activation

There was a similar (*p* > 0.05 for all) transient rise in neutrophils following all exposures, that had returned to baseline by 24 h post exposure (Supplement Table S2). However, there were no differences between exposures. Mean platelet activation did not differ between exposures, with mean platelet CD40 ligand and P-selectin expression and platelet-monocyte binding all being similar at 2 and 4 h post exposure, (p > 0.05 for all, Supplement Table S3).

### Fraction of exhaled NO

There were no significant changes in FE_NO_50 following exposures or differences comparing respective exposures (Supplement Table S4).

## Discussion

To our knowledge, this study represents the first detailed investigation of the impact of biodiesel exhaust emissions on functional cardiovascular endpoints (vasomotion, thrombus formation, fibrinolytic markers, platelet function and blood cell differential including inflammatory cells) in healthy human volunteers. The study also performed a detailed characterization of the tail-pipe emissions, with physiochemical profiling of the PM within the exhaust. We have shown that, despite chemical and physical differences, the acute effects of biodiesel exhaust inhalation on thrombus formation and the vascular system were similar to those observed upon PDE exposure.

Numerous controlled experimental exposures studies in human volunteers have shown that exposure to PDE causes acute cardiovascular and respiratory effects. Acute exposure has consistently been shown to induce endothelial dysfunction, in terms of impaired vasomotor function and endogenous fibrinolysis [7, 8, 23–25, 27]. Inhalation of PDE also induces thrombus formation [8, 23, 26] platelet activation [7, 8, 23], arterial stiffness [9] as well as systemic and airway inflammation [20, 28–31]. Exposure to fine PM of concentrated ambient urban particles (CAPs) has also been found to increase blood pressure and arterial vasoconstriction [32].

The vascular effects of PDE compared with filtered air exposures have been highly consistent in previous studies from our group. Specifically, when compared with filtered air exposures, PDE exposures have resulted in impairment of vasomotor function along with increased blood thrombogenicity and platelet activation [7, 8, 23–27]. In Fig. S1 we display the clear and consistent vasomotor dysfunction caused by exposure to PDE vs. filtered air based on pooled data from four historical studies using similar set ups [23–26]. Performing experimental studies with a three arm design with exposures, canulations and infusions is logistically and practically very difficult to perform, according to our experiences [26]. We therefore decided not to replicate the well-established vasomotor effects of PDE exhaust vs. filtered air (Fig. S1), but prioritized a direct comparison of the effects of PDE exhaust with RME biodiesel exhaust, in subjects exposed in the same facility and assessed by similar methods as previously. In contrast to vascular and thrombotic measures, our previous studies showed only modest and variable effects on secondary endpoints such as systemic inflammatory responses in terms of blood cells, soluble markers of inflammation and exhaled NO. By using an identical exposure setup and study design as in our previous experimental exposure studies, we have, for the first time, been able to show that replacing petrodiesel with RME30, or RME100, does not substantially alter the vascular response compared to exposure to conventional PDE exhaust. Cardiovascular outcomes (blood flow responses and thrombus formation) were similar for PDE and RME30 under exposure conditions that generated similar particle mass concentrations and, for PDE and RME100, when emission factors were held constant. Thus, these findings imply that the emissions from the RME employed would have comparable adverse effects on cardiovascular health as conventional PDE.

Biodiesel differs from petrodiesel in chemical composition, as it consists mainly of fatty acid methyl esters, whereas petrodiesel mainly contains saturated aliphatic hydrocarbons and aromatics. This gives biodiesel a higher cetane number, meaning it ignites faster [33]. It has also an increased ratio of oxygen, which lowers energy density and, at the same time, increases oxygen content in the ignited fuel spray. Increased oxygen content (which reduces soot formation) together with lower content of aromatics (soot precursors) and sulfur act together to decrease particulate emissions from biodiesel compared to petrodiesel [33]. Consistent with our findings, previous studies have demonstrated that increased biodiesel content in blends with petrodiesel fuel, is associated with reduced PM mass and increased NOx [20, 34, 35]. In addition, previous studies have indicated that addition of biodiesel to conventional petrodiesel fuel is associated with reduced CO emissions and a higher organic fraction and, in contrast to the present study, reduced hydrocarbons [36]. In RME30, exhaust concentrations of PM-bound PAHs were considerably lower compared to petrodiesel, in line with previous studies [37].

We suggest that it is unlikely that the minor differences in gaseous components, such as NO_x_, would play a significant role eliciting the acute cardiovascular effects, as previous exposure studies of the gaseous components alone, or of pure NO_2_ at similar concentrations, did not result in any acute cardiovascular impairment [26, 27, 38].

In the present study, the substitution of petrodiesel with a 100% RME formulation.

reduced the PM mass concentration when emission factors were held constant, but slightly increased the particle number concentration, along with a shift in the size distribution towards the ultrafine range (< 100 nm). We demonstrated a shift from a mono-modal distribution with a peak around 100–140 nm for the PDE exposures to a bi-modal distribution with one peak around 80–90 nm and a second larger peak around 30–40 nm during the RME100 exposures. In previous studies, there has been some inconsistency when it comes to size distribution of PM in biodiesel exhaust compared to PDE. Several reports support a similar shift towards smaller particles and an increase in total particle number in RME exhaust [34, 39–41], whereas others have found decreased particle number concentrations [42]. It has been suggested that the ultrafine (< 100 nm) mode of RME exhaust may be composed either of organic matter from RME fuel residues or production constituents with high boiling points, such as triglycerides and glycerol, or of inorganic fuel constituents and/or impurities such as alkali metals, metalloids and trace metals, which may act as condensation nuclei for condensable organics [41]. From our results, it is not possible to draw any conclusions regarding the composition of the different particle size modes. Though, still the subject of debate, it has been proposed that PM in the ultrafine range may be a major contributor to the adverse health effects following inhalation, due to greater surface area relative to mass of PM and higher deposition in the alveoli [43, 44]. In relation to health effects, particle size remains a complex issue, which is yet to be resolved and it remains speculative to suggest to what extent the shift in particle size influenced any endpoints in the current studies.

In our study, we found the oxidative potential of PM from RME exhaust to be lower than that of PM from PDE. Others have suggested RME-derived PM to have similar, or less, oxidative potential than petrodiesel exhaust [45], whilst soy-derived biodiesel PM may have increased oxidative potential (measured as dithiothreitol consumption) compared to petrodiesel-derived PM on a per mass basis [46]. This inconsistency may reflect that biodiesel exhaust in general, and particle properties in particular, is highly variable due to different fuel sources, formulations, storage conditions and combustion properties and engine conditions [13, 47, 48].

Oxidative stress is thought to be a key underlying mechanism driving inflammation following PM exposure [3, 44, 49]. We demonstrated a mild transient systemic inflammation, in terms of increased neutrophil counts, that was similar following all exposures over the observation period. In a study investigating the relative toxicity of PM from different fuels on mouse macrophages, Jalava et al. reported dose-dependent inflammation responses that were similar, or slightly smaller, for RME compared to petrodiesel-derived PM. This trend was stronger with catalytic after-treatment of the exhaust, or when the emission factor was taken into account, as RME produced less PM [45]. Oxidative stress can also result in DNA damage and, in previous in vitro studies, RME-derived PM has been shown to increase mutagenicity and cytotoxicity [42, 48, 50] compared to PDE. Complementary techniques, such as omics approaches, have potential to elucidate mechanism further, but were not included in this study [51]. Cell based techniques may also further delineate the complexity of the oxidative processes.

In this study, we aimed to assess the cardiovascular effects of biodiesel/biodiesel blend, as an alternative fuel to petrodiesel. Given the aim and that the findings of diesel exhaust-induced endothelial dysfunction, in terms of blunted vasomotor function and impaired endogenous fibrinolysis, together with increased ex vivo thrombus formation, are highly reproducible and consistent in a long series of studies [7–9, 23–27], we did not include a filtered air exposure. The addition of an air exposure would also have added substantially to the complexity of the study, demanding exposures and invasive vascular investigations, three times in each participant. While there at present is no directly comparable controlled exposure study in human subjects investigating detailed biodiesel-induced cardiovascular endpoints, the study by Mehus and coworkers provides some opportunities for comparisons [52]. That study was performed in a mining environment and compared health endpoints in human subjects from a pre-exposure day with measurements carried out in the aftermath of two separate exposures to PDE and to a biodiesel blend (75% soybean methyl ester (SME) with 25% PDE). The data indicated slightly reduced lung function, increased inflammatory markers in peripheral blood and induced sputum, which did not differ between the petrodiesel and SME 75% biodiesel exhaust exposures [20]. No filtered air exposure day was included, reflecting the complexity of performing exposure studies of health effects in human volunteers.

### Limitations of the study

The study was not gender balanced, which means the findings cannot be inferred to be applicable in females as well. Our series of earlier studies has, however, not indicated any sex related differences in the vascular response to diesel exhaust.

It is also recognized that subjects with pre-existing disease such as cardiovascular or respiratory diseases have not been studied and could potentially be a more sensitive and reactive population. The subjects were only studied after one single exposure, whereas in real life repeat exposures could potentially elicit enhanced responses. Respiratory effects were addressed to a limited extent in this study and should be explored in more detail in further studies. These aspects all warrant further research into health effects of exhaust from renewable engine fuels in different settings and groups.

## Conclusion

In healthy subjects, exhausts from the two biodiesel formulations RME30 and RME100 were not shown to have significantly different effects on any investigated marker of vascular function or thrombus formation compared to PDE, despite contrasting physico-chemical properties. We suggest that replacing petrodiesel with RME30 or RME100 does not alleviate acute cardiovascular impairment following exhaust inhalation, even in light of reduced PM mass. Whilst there are potential environmental benefits of introducing biofuels, the effects on human health should be a strong consideration when the future use of alternative fuels are determined.

## Methods

The study assessed the acute vascular and thrombotic effects of replacing petrodiesel with a 30% or 100% rapeseed biodiesel formulation, following controlled exposure in healthy human subjects. It was hypothesized that inhalation of exhaust from biodiesel formulations would induce less vascular effects compared to standard petrodiesel fuel.

### Study design

Two separate randomized controlled double-blind crossover studies were conducted. In study one, 16 subjects (14 male) were exposed both to PDE and exhaust generated from a biodiesel blend of 30% RME and 70% petrodiesel (RME30) on separate occasions. For females, the exposures were carried out in the same phase of the menstrual cycle. In study two, 19 subjects (all male) were separately exposed both to exhaust from 100% RME biodiesel without blending with petrodiesel (RME100) and PDE. In both studies, exposures were allocated in random order at least one week apart. Exposures were blinded for the subjects and researchers involved, but known to the engineer running the exposure facility from an adjacent room. Investigators were unblinded to the exposure codes only after completion of the full statistical analyses.

Exposures lasted one hour with intermittent exercise on a bicycle ergometer for 15 min alternating with 15 min rest. The bicycle ergometer was calibrated to give a minute ventilation during exercise of 20 L/m^2^ body surface area, as determined during a pre-test day. Exposures were conducted at the same time in the mornings in a purpose-built human exposure chamber, as described previously [23]. Study 1 was performed April–June with an interval of 7–50 days (mean 20 days) between exposures and study 2 September–December with a 7–46-day interval (mean 39 days). Subjects were not allowed to take any medication, supplemental vitamins or antioxidants during the week before and during the study and instructed to take a light low nitrate breakfast on exposure days, but otherwise to maintain their normal diet. At mid-day, subjects were given a standardized snack consisting of a protein drink and a piece of fruit. No other food or drink other than water was allowed during the measurements.

### Study population

Thirty-six healthy subjects (34 men and 2 women, aged 20–38 years, mean 27 years) were recruited, according to Good Clinical Practice (GCP) principles and as approved by the regional ethical review board and the study was performed in accordance with the Declaration of Helsinki, with the written informed consent from all participants. All volunteers were never regular smokers. A history of limited occasional social smoking was allowed, but not within the last 6 months. They underwent a physician interview and physical examinations and were determined to have normal physiologic parameters, body mass index (BMI), lung function, blood chemistry and electrocardiogram, according to the normal values of the hospital. Exclusion criteria were diabetes mellitus, cardiovascular disease, asthma, smoking or snus usage (powdered tobacco used orally). Female subjects took a urinary pregnancy test before each exposure to exclude pregnancy and subjects currently using birth control were excluded. The subjects were all healthy without any current disease or prescribed medication, including non-steroidal anti-inflammatory drugs (NSAID). All were instructed to maintain their normal diet, with no major changes, or additional intake of antioxidant rich components. The imbalance of sex in the study was unintentional and depending on responses to participate in the study: As a consequence, study data are mainly related to male gender.

### Exhaust exposure

RME100 biodiesel and low-sulfur standard diesel (petrodiesel) were acquired from Preem AB (Stockholm, Sweden). RME30 was blended on site using RME100 and petrodiesel. The RME30 contained the proprietary fuel additive ACP (Active Cleaning Power) according to instructions from the fuel company. The additive has been indicated to improve combustion and decrease engine deposits and is intended to be commercially available. The exact chemical composition is confidential, however, Preem AB has disclosed it to consist of detergent, lubricant and cetane-number improving agents, given as personal information to a lead engineer at the SMP engineering site running the engine set up.

Exhaust was generated using a Volvo diesel engine (Volvo TD40 GJE, 4.0 L, 4 cylinders) running under variable load, according to the urban part of the European Transient Cycle [25]. This engine represents an older generation of diesel engines, that are still widely used, as these engines are extremely durable with slow turnover time from a global perspective. More than 90% of the exhaust was shunted away and the remainder was mixed with filtered air and fed into the exposure chamber to generate a target PM_10_ (PM with a mean aerodynamic diameter of < 10 μm) concentration of 300 μg/m^3^ for both PDE and RME30 [28]. For the RME100 exposure, an identical driving cycle was used with the same load and rounds per minute (rpm) pattern, without modifications to the engine, exhaust dilution system or exposure chamber. The study was specifically designed to investigate the health effects of the exhaust from a specific vehicle when replacing PDE with RME under the same driving conditions rather than similar PM mass concentration in the chamber for the exposures.

### Emission characterization

PM_10_ concentration was determined gravimetrically using PTFE filters (Pall Teflo Life Science 47 mm, 2 μm). Real-time measurement of PM_10_ with a tapered-element oscillating microbalance (Rupprecht & Patashnik, Albany, New York, USA) was used during exposures to achieve steady PM levels at 300 μg/m^3^ [25]. A scanning mobility particle sizer system (SMPS TSI, Shoreview, Minnesota, USA) was used to determine fine particle number concentration and size distribution (18–638 nm), which included an electrostatic classifier platform (TSI 3080, TSI GmbH) with a Differential Mobility Analyzer (TSI DMA 3081) and an ultrafine Condensation Particle Counter (TSI CPC 3025A). In addition, PM was also sampled by a Dekati Gravimetric Impactor (DGI) for subsequent analysis of metals and oxidative potential. The DGI classifies particle size according to aerodynamic diameter (cut-points; 2.5, 1.0, 0.5 and 0.2 μm). 47 mm PTFE plates were used as impactor substrates and 70 mm PTFE filters as back-up filters (< 0.2 μm). Exposure concentrations were kept as constant as possible and samples were collected at intervals to reflect the average exposure scenarios.

For the carbon fractionation, standard 47 mm tissue quartz filters and 47 mm PTFE membrane filters were used. The analysis of particulate carbon fractions was performed by a standard thermal-optical method to determine the content of organic carbon (OC) and elemental carbon (EC) applying the EUSAAR 2 thermal protocol and a thermal-optical carbon analyzer (Sunset Laboratory Inc., Portland, Oregon, USA).

### Assessment of metal content in exhaust particulate

Metal content was assessed using an ELAN DRC ICP-MS (MSF008). The three last stages of the DGI sampler were used, i.e. filters; PM_< 0.2_, PM_0.2–0.5_ and PM_0.5-1_μm and the extraction was performed in HPLC-grade methanol, followed by vortexing and sonication in a water bath (max power, Clifton SW12h, Nickel-Electro Ltd., Weston-super-Mare, UK). The removed filter was then rinsed in methanol. Particle extract was subsequently dried down under nitrogen and recovered mass determined. Mass recoveries for the PM_< 0.2_ and PM_0.2–0.5_ μm filters were good; 84.0 ± 21.3% (filter loadings ranging from 510 to 860 μg) and 113.0 ± 38.7% (170–370 μg) respectively. Recoveries from the PM_0.5–1.0_ μm filters were poor, reflecting the low filter loadings (20–180 μg) and were not used for subsequent analysis. Following particle resuspension in 1.0 mL of Chelex resin-treated water (sonication at max power), 100 μL aliquots were added to dilute Aqua Regia, spiked with an internal standard containing 1 ppm indium (isotope 115), gallium (69), bismuth (209) and yttrium (89), sealed in Teflon vessels and placed in a 90 °C water bath. The mixture was cooled prior to the addition of Chelex-resin treated water. Metals quantified were: aluminum [26], arsenic (75), barium (135), beryllium [9], manganese [53], vanadium [51], antimony (121), cadmium (111), chromium [54], copper (63), molybdenum (95), nickel (60), lead (208), zinc (66), strontium (88), calcium [45], iron (56), boron [11], cobalt (59), rubidium (85), cesium (133), gold (197). Elemental concentrations were determined with reference to a 6-point standard curve based on an ICP multi element standard solution VI CertiPUR® (Merck, Lot. No. OC529648) or, where not present, in the multi-elemental standard the elements own standard curve (ICP standard, MERCK).

### Measurement of PAHs in exhaust

For collection of particulate and semi-volatile PAHs, sampling with 47 mm glass fiber filters followed by 70 mm polyurethane foam plugs (PUFs) were performed in the chamber during the exposures. Both filters and polyurethane foam plugs (PUFs) were spiked with an isotope labeled internal standard mixture. The samples were extracted with pressurized liquid extraction using an ASE 200 Accelerated Solvent Extractor system (Dionex Corporation, Sunnyvale, CA, USA). PUFs were extracted using hexane at 100 °C and 1100 psi (7.58 MPa). Particles were extracted with toluene/methanol 9:1 (v/v) at 200 °C and 3000 psi (20.7 MPa).

The raw extracts were concentrated to approximately 0.5 mL followed by solid phase extraction (SPE), and the SPE-purified extracts were analyzed using an in-house built HPLC-GC-MS system [54]. Data were acquired and processed using MSD ChemStation (Agilent Technologies, Santa Clara, California, USA). PAHs were quantified using single point calibration. Compound identity was determined through compound specific mass to change ratio and its relative retention time on the GC capillary column.

Reported concentrations of semi-volatile PAHs are the sum of; phenanthrene, anthracene, 3-methylphenanthrene, 2-methylphenanthrene, 2-methylanthracene, 9-methylphenanthrene, 1-methylphenanthrene, 4*H*-cyclopenta [*def*] phenanthrene, 2-phenylnaphthalene, 3,6-dimethylphenanthrene, 3,9-dimethylphenanthrene, fluoranthene, pyrene, 2-methylpyrene, 4-methylpyrene, 1-methylpyrene, benz [*a*] anthracene, and chrysene. Reported concentrations of PM associated PAHs include those measured in the semi-volatile phase and the sum of benzo [*b*] fluoranthene, benzo [*k*] fluoranthene, benzo [*e*] pyrene, benzo [*a*] pyrene, perylene, indeno [1,2,3-*cd*] pyrene, and benzo [*ghi*]perylene.

### Assessing oxidative potential of PM

Oxidative potential of PM was first determined by electron paramagnetic resonance (EPR; or electron spin resonance) by measuring oxidation of a spin-trap with preferential selectivity for superoxide free radicals [55]. Briefly, 47 mm teflon filters with PM were suspended in a physiological saline solution (Krebs buffer) at a particle concentration of 100 μg/mL Samples were vortexed, followed by sonication (100% power; Fisherbrand FB11002; Fisher Scientific, Loughborough, UK). Suspensions were incubated with the spin-trap, Tempone-H (1 mM; Enzo Life Sciences, Exeter, UK), for 60 min before measurement. Pyrogallol (100 μM), a spontaneous generator or superoxide radicals, was used as a positive control. An X-band EPR spectrometer (Magnettech MS-200, Berlin, Germany) was used. Baseline signals from blank filters were subtracted from that of filters with particulate.

Oxidative potential of PM was also determined by measuring glutathione levels in a synthetic respiratory tract lining fluid (sRTLF) following in vitro exposure to PM. Extracted PM (50 μg/mL) was incubated in sRTLF containing physiologically relevant concentrations of urate (UA), ascorbate (AA) and glutathione (GSH), adjusted to pH 7.0 and incubated at 37 °C for four hours. Samples were centrifuged to remove particles, prior to samples acidification with metaphosphoric acid (final concentration 5% w/v). The remaining concentrations of ascorbate and urate were quantified using reverse phase HPLC with electrochemical detection (Jones Chromatography, Hengoed, Wales). Glutathione concentrations were determined with the GSSG-reductase-5,5′-dithio-bis(2-nitrobenzoic acid) (DNTB) recycling assay. Full details of the methodology and derivation of the OP metrics have been described previously [53, 56].

### Ex vivo blood coagulability model

Ex vivo thrombus formation was assessed two hours post-exposure using a Badimon chamber perfusion model of acute arterial injury as described previously [8]. In brief, a venous cannula is inserted into an antecubital vein and blood drawn using a peristaltic pump at a rate of 10 mL/s via tubing connected to a perfusion chamber submerged in a water bath at 37 °C. This model of acute arterial injury consists of three consecutive chambers containing porcine aortic strips from which the intima is removed, to exposure a prothrombotic surface that simulates deep arterial injury following plaque rupture. Blood is passed through the chambers for five minutes, followed by rinsing with saline. The strips are removed, fixed in paraformaldehyde, embedded for histological analysis, then cut and stained with Masson’s trichrome. The sections are analyzed using a semi-automatic microscope and total thrombus area quantified [8].

### Vascular endothelial function

Vascular vasomotor function was assessed using forearm venous occlusion plethysmography four to six hours after each exposure, as described previously [23–25, 27]. The brachial artery was cannulated with a 27-standard-wire-gauge steel needle. Following a saline infusion, four vasodilator drugs, reflecting NO-dependent and non-NO-dependent (verapamil) vasodilation, were infused separately at 1 mL/min in incremental doses separated by washouts to restore baseline flow. Bradykinin (100, 300 and 1000 pmol/min); acetylcholine (5, 10 and 20 μg/min); sodium nitroprusside (2, 4 and 8 μg/min) were infused in random order, and verapamil (10, 30 and 100 μg/min) was infused last due to its long acting effects. Mercury-in-silicone strain gauges were used to assess forearm blood flow in both arms (infused arm and non-infused arm) simultaneously. Bradykinin induces the release of the fibrinolytic mediator, tissue plasminogen activator (t-PA) from the vascular endothelium. t-PA antigen concentrations were determined in blood samples collected before the first and following each dose of bradykinin, using enzyme-linked immunosorbent assay (TintElize tPA, Biopool EIA, Trinity Biotech, Bray, Ireland).

### Blood cell counts

Venous blood samples for blood cell counts were obtained pre-exposure and 2, 4, 8 and 24 h post-exposure and analyzed at an accredited hospital laboratory.

### Assessing platelet activation with flow cytometry

Platelet activation was assessed using flow cytometry at 2 and 4 h post-exposure. Whole blood samples were immediately labeled with monoclonal antibodies (mAbs) for flow cytometric analysis: CD14-conjugated phycoerythrin (PE) (DAKO, Glostrup, Denmark) specific for monocytes, fluorescein isothiocyanate (FITC)-conjugated CD42a (Serotec, Oxford, UK) specific for platelets and isotype-matched control (Serotec, Oxford, UK). CD14-FITC and CD40-PE (Serotec, Oxford, UK) were used for analysis of CD40 positive monocytes. After incubation, cells were fixed and lysed with FACS-Lyse solution (Becton Dickinson, Franklin Lakes, New Jersey, USA) and samples were analyzed using Facs Calibur flow cytometer (Becton Dickinson, Franklin Lakes, New Jersey, USA). Platelet-monocyte aggregation and CD40 positivity of monocytes were expressed as percentage of 2000–2500 collected monocytes. In addition, to determine platelet surface expression of P-selectin and CD40-ligand, whole blood was labeled with CD42a (FITC), CD62p (PE) specific for P-selectin, CD154 specific for CD40 Ligand and isotype controls. Cells were analyzed with a Facs Calibur flow cytometer. Expression of P-selectin and CD40L were expressed as percentage of collected number of platelets (7500).

### Respiratory measures

Forced expiratory volume during first second (FEV_1_) and vital capacity (VC) were measured at baseline and 8 h post-exposure by spirometry (Jaeger Masterlab, Carefusion, San Diego, California) according to ATS/ERS guidelines (56). Fraction of exhaled NO (FE_NO_) concentrations were evaluated using a nitric oxide analyzer (NIOX®, Aerocrine AB, Stockholm, Sweden) at an exhalation rate of 50 mL/s (± 10%), FE_NO_50. Lung function measurements post-exposure were performed at 8 h based on findings from preceding studies as well as logistic aspects. As vascular measurements were prioritized in the current study lung function was mainly included to rule out major respiratory effects.

### Data analysis and statistics

Based on our previous studies of endothelial vasomotor function and endogenous fibrinolysis, to detect a 20% difference in forearm blood flow and a 16% difference in t-PA release, we require sample sizes of *n* = 16 at 80% power and two-sided *P* < 0.05. Statistical analyses were performed with GraphPad Prism (version 5, GraphPad Software, La Jolla, California, USA). Data were analyzed using paired and unpaired Student’s t-test, Wilcoxon signed rank test and repeated measures analysis of variance (ANOVA), as appropriate. For blood cell counts, post-exposure changes from baseline were calculated at individual level. Statistical significance was taken at 2-sided *p* < 0.05.

## Supplementary Information

**Additional file 1.**

**Additional file 2.**

## Data Availability

All relevant data are included in the manuscript and supporting information. These are also available from the authors upon reasonable request.

## Abbreviations

ACP: Active Cleaning Power
CAPS: concentrated ambient particles
EC/OC ratio: elemental carbon/organic carbon ratio
EPR: electron paramagnetic resonance
FE_NO_50: Fraction of exhaled nitric oxide at 50 L/min flow
OP^AA^: oxidative potential for ascorbic acid
OP^GSH^: oxidative potential for glutathione
OP^TOT^: total oxidative potential
PAHs: Polyaromatic hydrocarbons
PDE: 100% petrodiesel fuel exhaust
PM: Particulate matter
PM_10_: PM with a mean aerodynamic diameter of < 10 μm
PM_2.5_: PM with a mean aerodynamic diameter of < 2.5 μm
RME 30: Rapeseed methyl ester 30% blended diesel fuel
RME 100: Rapeseed methyl ester 100% exhaust
