# Supplementary material for: Acute cardiovascular effects of controlled exposure to dilute Petrodiesel and biodiesel exhaust in healthy volunteers: a crossover study
Source: Part Fibre Toxicol. 2021 Jun 14;18:22. doi: 10.1186/s12989-021-00412-3 (PMC8204543; doi:10.1186/s12989-021-00412-3)
Supplement: Supplementary file 1 — Additional file 1. [file 12989_2021_412_MOESM1_ESM.pdf]

Supplemental Material

Acute Cardiovascular Effects of Controlled Exposure to Dilute Petrodiesel and Biodiesel Exhaust Emissions in Healthy Volunteers: A Crossover Study

Table S1

Total metal and metalloid concentrations measured in $PM_{<0.2}$ and $PM_{0.2\text{-}0.5}$ fractions derived from petrodiesel and RME blended fuels.

Element	Petrol diesel		RME30		RME100	
(µg/mg)	$PM_{<0.2}$	$PM_{0.2\text{-}0.5}$	$PM_{<0.2}$	$PM_{0.2\text{-}0.5}$	$PM_{<0.2}$	$PM_{0.2\text{-}0.5}$
^{27}Al	0.38±0.36	0.70±1.19	1.35±2.33	0.08±0.13	7.56±10.18	0.19±0.33
^{75}As	0.01±0.01	0.05±0.01	0.00±0.00	**0.00±0.00**	0.00±0.00	0.05±0.05
^{135}Ba	0.03±0.02	0.04±0.04	0.03±0.04	0.03±0.02	0.06±0.03	0.02±0.02
^{55}Mn	0.23±0.22	0.47±0.30	2.31±2.49	**3.96±2.14**	2.23±2.95	**6.98±3.59**
^{50}V	1.15±0.99	4.57±3.92	27.00±28.56	**47.82±23.10**	26.39±37.60	**89.21±48.50**
^{52}Cr	1.23±1.08	5.01±4.31	29.47±31.59	**50.82±25.40**	28.61±40.67	**96.35±52.97**
^{63}Cu	0.25±0.19	0.81±0.59	0.24±0.17	**0.38±0.12**	0.68±0.53	0.77±0.41
^{95}Mo	0.02±0.01	0.05±0.03	0.33±0.35	**0.63±0.35**	0.28±0.40	**0.92±0.50**
^{60}Ni	1.09±0.73	3.00±2.43	15.07±16.51	**25.12±12.86**	14.52±20.31	**48.03±25.89**
^{66}Zn	2.61±0.48	4.83±2.08	3.47±0.66	2.25±0.69	2.48±1.23	3.04±0.62
^{44}Ca	5.71±1.19	13.42±5.20	2.46±1.24	1.79±1.44	9.46±8.10	10.55±3.48
^{56}Fe	5.45±4.19	19.21±15.00	106.51±114.62	**180.76±101.44**	96.29±131.28	**305.33±145.50**
^{11}B	0.31±0.13	0.97±0.88	**0.00±0.00**	0.00±0.00	**0.07±0.12**	0.07±0.12
^{197}Au	0.14±0.16	0.40±0.49	0.00±0.00	0.00±0.00	0.02±0.04	0.00±0.00
^{88}Sr	0.02±0.00	0.04±0.02	**0.01±0.00**	**0.01±0.00**	0.02±0.01	0.02±0.02
^{59}Co	0.02±0.02	0.03±0.02	0.20±0.21	**0.35±0.19**	0.18±0.25	**0.58±0.3**
^{208}Pb	0.03±0.01	0.06±0.04	0.02±0.01	0.01±0.01	0.04±0.05	0.02±0.01
^{85}Rb	0.01±0.01	0.01±0.01	0.00±0.00	0.00±0.00	0.01±0.01	0.01±0.00
(ng/mg)						
^{9}Be	0.00±0.00	0.29±0.49	0.21±0.19	0.35±0.61	0.27±0.26	0.40±0.69
^{121}Sb	2.20±1.25	1.54±1.49	2.07±2.71	1.41±1.48	2.48±1.70	4.82±2.72
^{111}Cd	0.00±0.00	0.00±0.00	3.30±5.72	2.57±3.93	0.00±0.00	0.00±0.00
^{133}Cs	0.09±0.07	0.16±0.12	0.03±0.05	0.00±0.00	0.14±0.14	0.10±0.04

Data represent the mean (SD) of measurements made on PM extracts from three separate PM filters collected during separate exposures. All values are expresses as µg/mg PM, with the exception of Be, Sb, Cd and Cs, which are expressed as ng/mg PM. Figures in bold illustrate significant difference ($P<0.05$) in concentration within a given fraction relative to Petrodiesel value. Shaded rows illustrate elements where elements were quantified following reaction with ammonia using the dynamic reaction cell of the ICP-MS to remove potential polyatomic interferences.

Table S2

Exposure conditions from earlier studies with bilateral forearm plethysmography after petrodiesel exhaust and filtered air in healthy human subjects.

Study (ref)	Mills et al 2005 (23)	Törnqvist et al 2007 (24)	Barath et al 2010 (25)	Lucking et al 2011 (27)
Engine mode	Idling	Idling	ETC	ETC
PM1 (ug/m^3)	300	300	250	320
NO_2 (ppm)	1.6	n/a	0.9	0.7
NOx (ppm)	6.1	n/a	7.5	6.4
THC (ppm)	4.3	n/a	1.2	0.91
Number of subjects	30	15	18	27

Data given as mean values. ETC=European Transient Cycle, urban part. THC= total hydrocarbons. n/a; data not available but was part of the same exposure series and indicated to be similar to Mills 2005. Combined plethysmography data are presented in Figure S1.

Table S2

Change in Blood Cell Counts

	Hours post exposure	Petrodiesel	RME30	p value: Exposure	p value: Time	Petrodiesel	RME100	p value: Exposure	p value: Time
Change in Leukocytes, $x10^9$/L	**2 h**	0.89 [0.21, 1.56]	0.77 [0.01, 1.45]	0.67	<0.01	0.56 [0.05, 1.17]	0.83 [0.33, 1.33]	0.28	0.02
	**4 h**	0.67 [0.10, 1.26]	0.77 [0.15, 1.39]			0.56 [0.01, 1.11]	1.00 [0.56, 1.44]		
	**8 h**	1.01 [0.31, 1.84]	1.27 [0.43, 2.11]			1.11 [0.31, 1.90]	0.62 [0.04, 1.20]		
	**24 h**	0.04 [-0.37, 0.45]	0.01 [-0.50, 0.48]			-0.06 [-0.34, 0.21]	-0.14 [-0.34, 0.24]		
Change in Lymphocytes, $x10^9$/L	**2 h**	-0.29 [-0.48, -0.11]	-0.18 [-0.40, 0.04]	0.71	<0.01	-0.32 [-0.60, -0.04]	-0.29 [-0.54, 0.04]	0.45	0.61
	**4 h**	-0.27 [-0.53, 0.02]	-0.23 [-0.43, 0.03]			-0.17 [-0.45, 0.10]	-0.07 [-0.32, 0.19]		
	**8 h**	0.03 [-0.22, 0.29]	0.07 [-0.09, 0.24]			-0.08 [-0.40, 0.23]	-0.09 [-0.33, 0.15]		
	**24 h**	0.14 [-0.10, 0.38]	0.09 [-0.10, 0.28]			-0.11 [-0.26, 0.04]	-0.14 [-0.28, -0.01]		
Change in Monocytes, $x10^9$/L	**2 h**	-0.08 [-0.14, 0.01]	-0.04 [-0.12, 0.04]	0.22	0.02	0.01 [-0.04, 0.05]	0.04 [-0.01, 0.09]	0.07	0.41
	**4 h**	-0.05 [-0.14, 0.01]	-0.02 [-0.07, 0.03]			0.00 -[0.05, 0.5]	0.03 [-0.03, 0.08]		
	**8 h**	0.01 [-0.08, 0.10]	0.03 [-0.07, 0.14]			0.03 [-0.03, 0.10]	-0.01 [-0.07, 0.06]		
	**24 h**	0.03 [-0.10, 0.05]	0.01 [-0.05, 0.06]			-0.11 [-0.26, 0.04]	-0.02 [-0.07, 0.03]		
Change in Neutrophils, $x10^9$/L	**2 h**	1.29 [0.73, 1.85]	1.04 [0.48, 1.60]	0.63	<0.01	0.89 [0.38, 1.41]	1.13 [0.62, 1.64]	0.27	<0.01
	**4 h**	1.05 [0.61, 1.49]	1.10 [0.49, 1.71]			0.76 [0.38, 1.13]	1.11 [0.73, 1.49]		
	**8 h**	1.09 [0.48, 1.70]	1.30 [0.37, 2.23]			1.19 [0.60, 1.78]	0.76 [0.40, 1.11]		
	**24 h**	-0.10 [-0.39, 0.19]	-0.05 [-0.43, 0.32]			0.07 [-0.21, 0.36]	0.14 [-0.03, 0.32]		
Change in platelets, $x10^9$/L	**2 h**	-11 [-19, -4]	-15 [-21, -9]	0.09	<0.01	-11 [-17, -7]	-9 [-16, -3]	0.67	<0.01
	**4 h**	-10 [-15, -4]	-16 [-21, -11]			-12 [-18, -7]	-8 [-17, 0]		
	**8 h**	-19 [-30, -8]	-18 [-25, -11]			-21 [-28, -15]	-24 [-39, -10]		
	**24 h**	3 [-5, 10]	-3 [-13, 7]			8 [2, 14]	8 [1, 16]		

Absolute change in number of cells x109/L in peripheral blood, compared to pre-exposure, mean with 95% CI. Left panel Petrodiesel vs. RME30 n=16. Right panel Petrodiesel vs. RME100 n=19. Changes in blood cell counts were similar after either exposure with a transient increase in leukocytes and neutrophils along with a reduction of platelets. All values returned to pre-exposure levels over 24 hours. P values for type of exposure and change over time from 2-way repeated measures ANOVA

Table S3

Platelet activation

	Hours post exposure	***Petrodiesel***	***RME30***	***P***	***Petrodiesel***	***RME100***	***P***
Platelet Monocyte Binding, %	2	35.5 [30.0, 40.9]	38.5 [31.7, 45.3]	0.43	36.4 [25.9, 46.9]	28.9 [20.3, 37.4]	0.15
	4	42.4 [36.3, 48.5]	39.9 [33.2, 46.6]	0.53	31.4 [22.7, 40.2]	27.8 [19.6, 35.9]	0.53
Monocyte CD40 positivity, %	2	18.3 [18.3, 19.9]	19.9 [12.8, 27.0]	0.53	3.8 [1.6, 6.0]	2.3 [0.6, 3.9]	0.25
	4	18.7 [11.6, 25.9]	17.4 [12.6, 22.2]	0.67	2.8 [1.3, 4.4]	2.3 1.2, 3.3]	0.46
Platelet CD40 Ligand Positivity, %	2	0.5 [0.4, 0.6]	0.5 [0.3, 0.6]	0.73	0.3 [0.0, 0.5]	0.2 [0.1, 0.2]	0.45
	4	0.4 [0.3, 0.5]	0.5 [0.3, 0.6]	0.81	0.2 [0.1, 0.3]	0.2 [0.1, 0.3]	0.93
Platelet p-selectin expression, %	2	1.0 [0.8, 1.3]	1.4 [0.8, 2.1]	0.18	0.5 [0.2, 0.8]	0.4 [0.1, 0.6]	0.31
	4	1.3 [0.8, 1.7]	1.0 [0.7, 1.2]	0.11	0.3 [0.2, 0.4]	0.4 [0.1, 0.6]	0.60

Platelet activation assessed with flow cytometry was similar after either exposure. Left panel Petrodiesel vs. RME30 n=16. Right panel Petrodiesel vs. RME100 n=19. P values from Student's t-test, mean with 95% CI.

Table S4

Fraction of exhaled NO

		Study 1				*Study 2*			
		Petrodiesel	***RME30***	***n***	***p***	***Petrodiesel***	***RME100***	***n***	***p***
FE_{NO}50 ml/s, ppb	**Pre exposure**	12.0 [8.9, 15,2]	11.1 [8.5, 13,7]	11		12.9 [9.9, 16.0]	15.3 [11.2, 19.3]	18	
	**8 h post exposure**	14.7 [10.1, 19.4]	13.3 [9.6, 17.1]	13	0.25	15.1 [11.4, 18.8]	14.6 [10.6, 18.6]	16	0.70

Fraction of exhaled NO at 50 ml/s, ppb. Mean values with 95% CI, p value for paired t-test 8 h post exposure.

Figure S1 (also given in separate file)

Legend:

Combined plethysmography data from four studies comparing the effects of petrodiesel with filtered air (ref 23, 24, 25, 27). Petrodiesel consistently causes vasomotor dysfunction to endothelial (acetylcholine, bradykinin, nitroprusside) and non-endothelial dependent vasodilators (verapamil) vs. filtered air. Technical and sampling difficulties during bilateral forearm brachial arterial needle infusions of vasoactive agents were reasons for not obtaining full sets of data from all individuals.

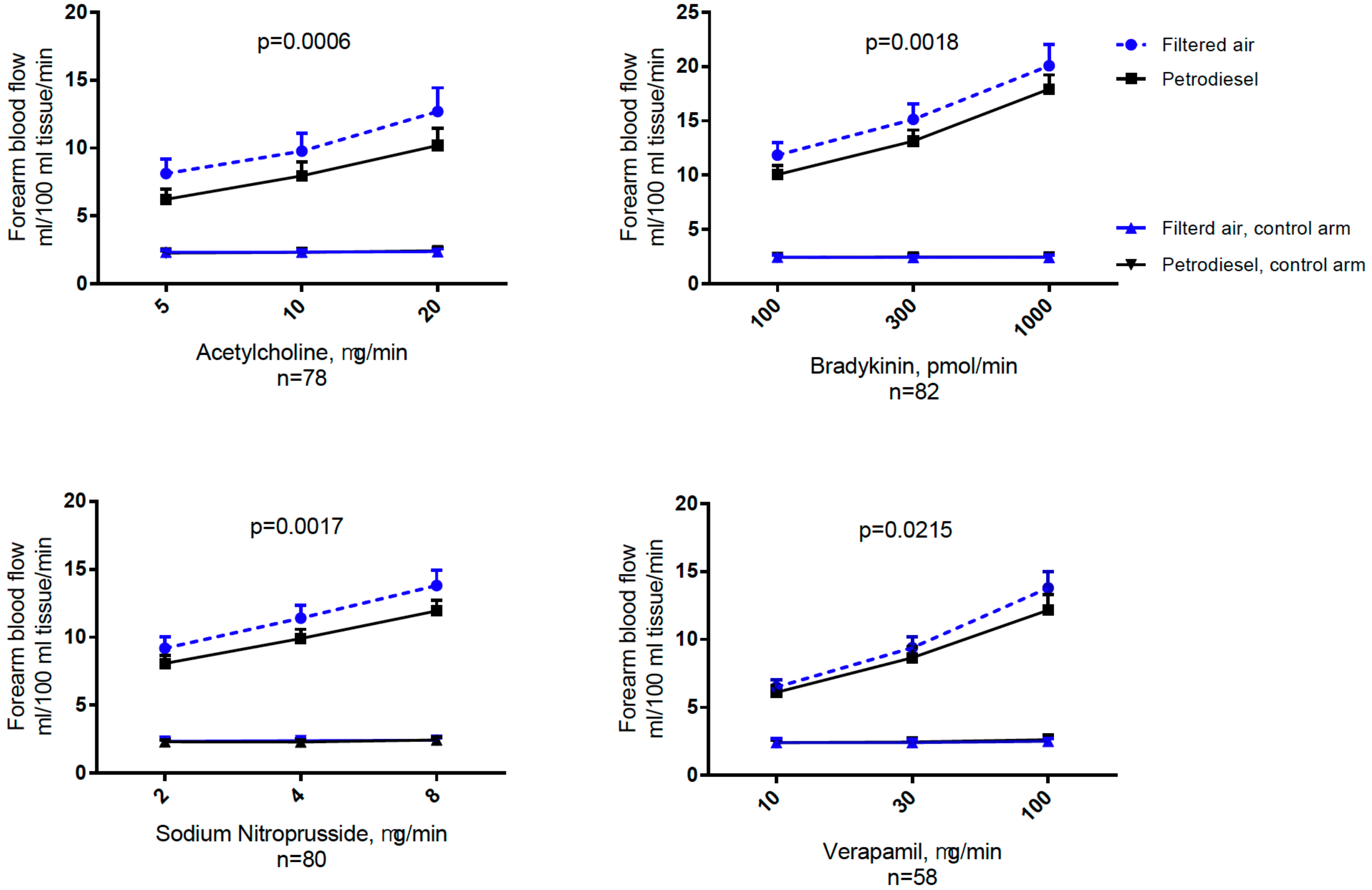
p=0.0006
Forearm blood flow
ml/100 ml tissue/min
Acetylcholine, mg/min
n=78
p=0.0018
Bradykinin, pmol/min
n=82
Filtered air
Petrodiesel
Filterd air, control arm
Petrodiesel, control arm
p=0.0017
Sodium Nitroprusside, mg/min
n=80
p=0.0215
Verapamil, mg/min
n=58
