# Supplementary figures and images for: Acute cardiovascular effects of controlled exposure to dilute Petrodiesel and biodiesel exhaust in healthy volunteers: a crossover study
Source: Part Fibre Toxicol. 2021 Jun 14;18:22. doi: 10.1186/s12989-021-00412-3 (PMC8204543; doi:10.1186/s12989-021-00412-3)

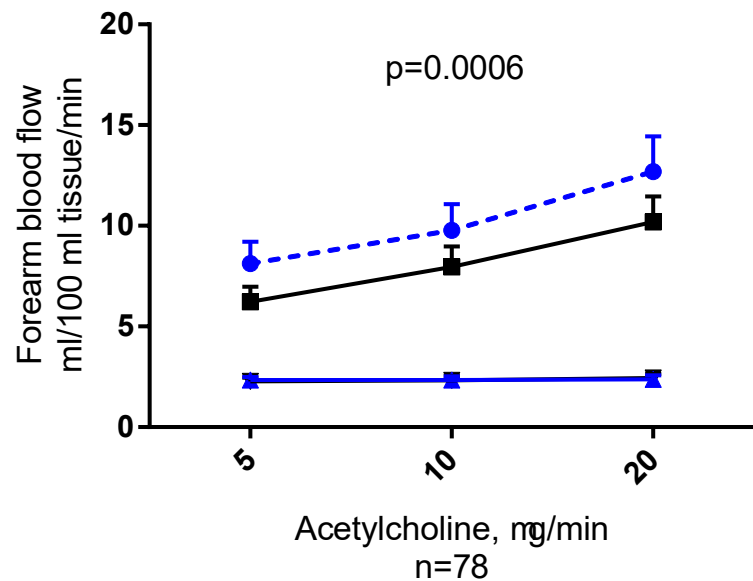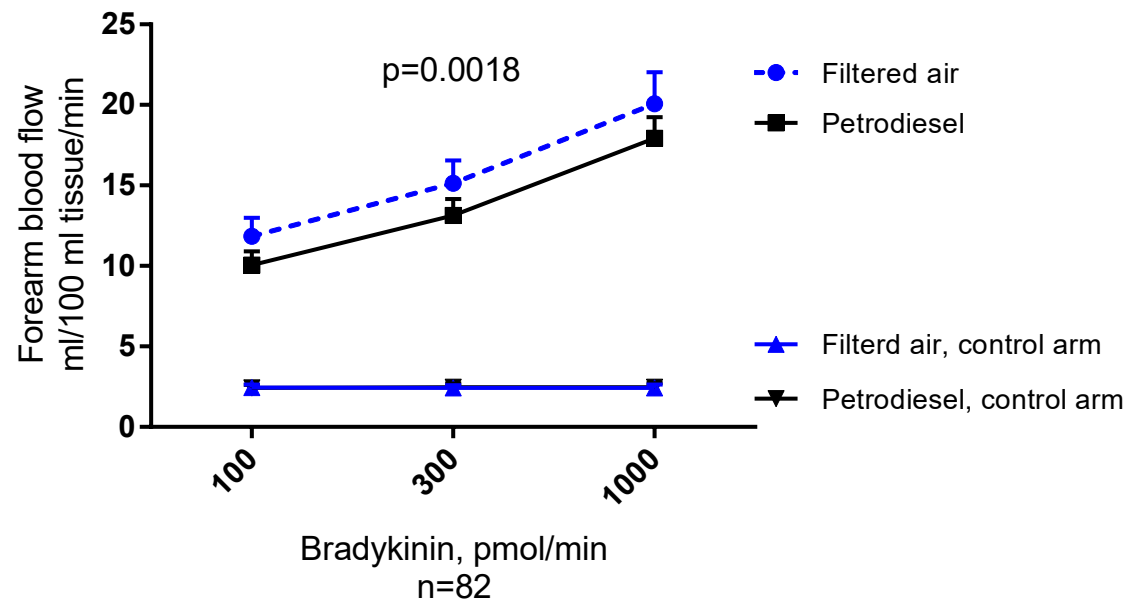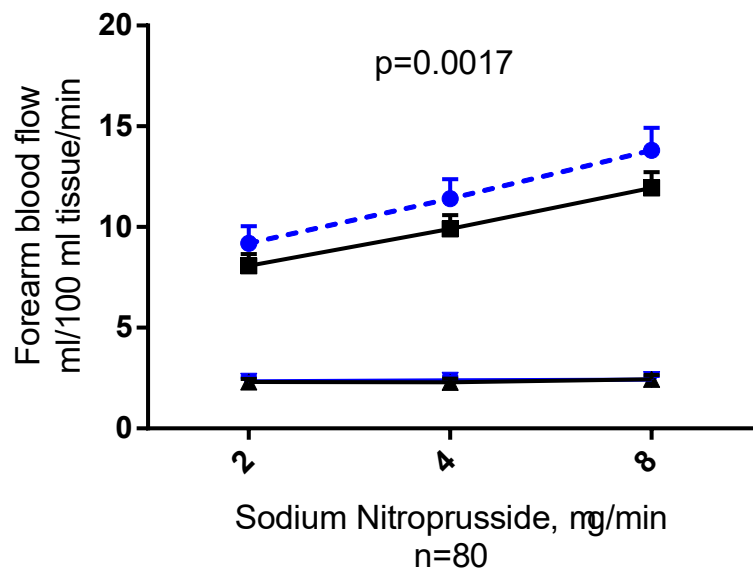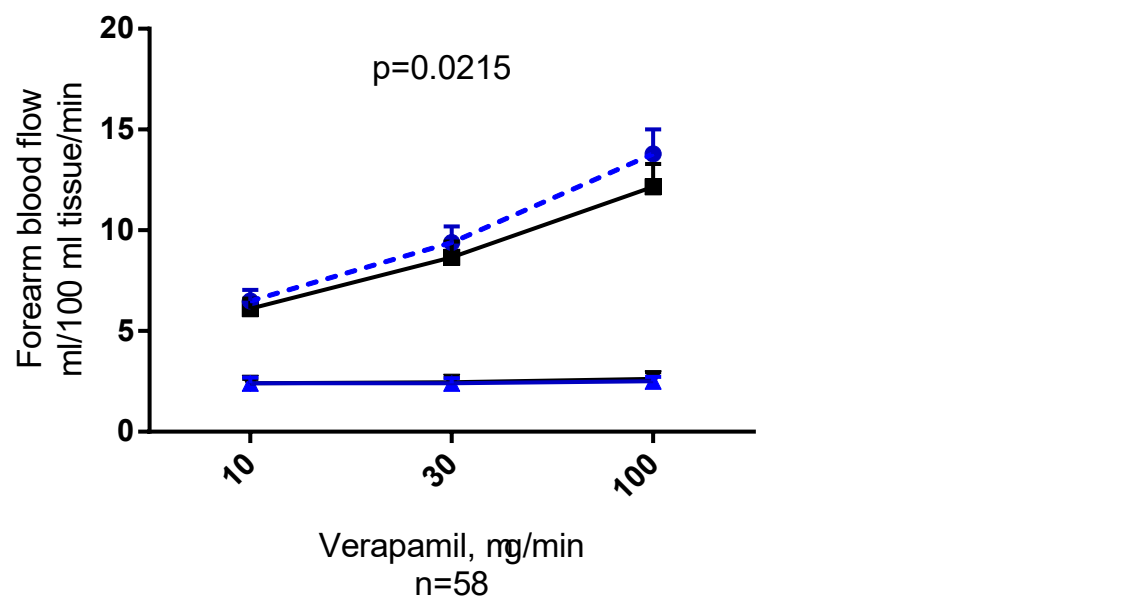

Supplement: Supplementary file 2 — Additional file 2. [file 12989_2021_412_MOESM2_ESM.pdf]
